# Postural instability and the condition of physical frailty in the elderly^*^

**DOI:** 10.1590/1518-8345.2655-3146

**Published:** 2019-04-29

**Authors:** Dayana Cristina Moraes, Maria Helena Lenardt, Marcia Daniele Seima, Bruno Henrique de Mello, Larissa Sayuri Setoguchi, Clarice Maria Setlik

**Affiliations:** 1 Hospital Nossa Senhora do Pilar, Unidade de Terapia Intensiva, Curitiba, PR, Brasil; 2 Universidade Federal do Paraná, Departamento de Enfermagem, Curitiba, PR, Brasil; 3 Prefeitura de São José dos Pinhais, Departamento de Atenção à Saúde, São José dos Pinhais, PR, Brasil; 4 Hospital Nossa Senhora das Graças, Unidade de Terapia Intensiva, Curitiba, PR, Brasil; 5 Bolsista da Coordenação de Aperfeiçoamento de Pessoal de Nível Superior (CAPES), Brasil; 6 Hospital Nossa Senhora das Graças, Unidade de Internação, Curitiba, PR, Brasil

**Keywords:** Postural Balance, Geriatric Nursing, Nursing, Frail Elderly, Dizziness, Vertigo, Equilíbrio Postural, Enfermagem Geriátrica, Enfermagem, Idoso Fragilizado, Tontura, Vertigem, Equilibrio Postural, Enfermería Geriátrica, Enfermería, Adultos mayores Fragilizados, Mareo, Vértigo

## Abstract

**Objective:**

to analyze the relationship between postural instability and the condition and markers of physical frailty of the elderly people in outpatient geriatric and gerontology care.

**Method:**

a cross-sectional study with a sample of 381 elderly subjects. Physical frailty was evaluated by the frailty phenotype and postural instability through the Berg Balance Scale. Univariate analyses consisted in Chi-square tests, and multivariate analyses used the *Forward Stepwise* method, which resulted in a model of physical frailty associated with postural instability.

**Results:**

among the participants, 56 (14.7%) were frail, 217 (57%) pre-frail, and 68 (28.3%) non-frail. Pre-frailty (*p* < 0.001), frailty (*p* = 0.000), and the markers hand grip strength (*p* = 0.0008), unintentional weight loss (*p* = 0.0094), level of physical activity (*p* = 0.0001), fatigue/exhaustion (*p* = 0.0001), and gait speed (*p* = 0.0001) were associated with postural instability.

**Conclusion:**

the presence of postural instability determines a greater chance of the elderly being frail or pre-frail. This result favors the planning of gerontological nursing care and strengthens the treatment plan under a specific approach.

## Abstract (pt)

**Objetivo:**

analisar a relação entre instabilidade postural e a condição e os marcadores de fragilidade física de idosos em assistência ambulatorial de geriatria e gerontologia.

**Método:**

estudo transversal com amostra constituída por 381 idosos. A fragilidade física foi avaliada mediante o fenótipo de fragilidade e a instabilidade postural pela Escala de Equilíbrio de Berg. Realizaram-se análises univariadas pelo teste *Qui*-quadrado e multivariadas pelo *Forward Stepwise*, que resultou no modelo de fragilidade física associado à instabilidade postural.

**Resultados:**

dos participantes, 56 (14,7%) eram frágeis, 217 (57%) pré-frágeis e 68 (28,3%) não frágeis. Associaram-se à instabilidade postural a pré-fragilidade (*p*<0,001), fragilidade (*p*=0,000) e os marcadores força de preensão manual (*p*=0,0008), perda de peso não intencional (*p*=0,0094), nível de atividade física (*p*=0,0001), fadiga/exaustão (*p*<0,0001) e velocidade da marcha (*p*<0,0001).

**Conclusão:**

a presença de instabilidade postural determina maior chance do idoso ser frágil ou pré-frágil. Esse resultado favorece o planejamento dos cuidados gerontológicos de enfermagem e fortalece o plano de tratamento sob uma abordagem específica.

## Abstract (es)

**Objetivo:**

analizar la relación entre inestabilidad postural, la condición y los marcadores de fragilidad física de adultos mayores en consultorio de geriatría y gerontología.

**Método:**

estudio transversal con muestra constituida por 381 adultos mayores. La fragilidad física fue evaluada mediante el fenotipo de fragilidad y la inestabilidad postural por la Escala de Equilibrio de Berg. Se realizaron análisis univariados con el test *Chi*-cuadrado y multivariados con el *Forward Stepwise*, que resultó en el modelo de fragilidad física asociado a la inestabilidad postural.

**Resultados:**

de los participantes, 56 (14,7%) eran frágiles, 217 (57%) pre-frágiles y 68 (28,3%) no frágiles. Se asociaron a la inestabilidad postural la pre-fragilidad (*p*<0,001), fragilidad (*p*=0,000) y los marcadores fuerza de prensión manual (*p*=0,0008), pérdida de peso no intencional (*p*=0,0094), nivel de actividad física (*p*=0,0001), fatiga/agotamiento (*p*<0,0001) y velocidad de la marcha (*p*<0,0001).

**Conclusión:**

la presencia de inestabilidad postural determina mayor chance del adulto mayor de ser frágil o pre-frágil. Ese resultado favorece la planificación de los cuidados gerontológicos de enfermería y fortalece el plan de tratamiento bajo un abordaje específico.

## Introduction

Frailty is considered a wide-ranging geriatric syndrome due to the high prevalence in the elderly population and the negative impact on the treatment plan of other geriatric syndromes, since frail elderly individuals are at increased risk for developing other disabilities^(^ ^1^ ^)^.

Experts on physical frailty define it as a “medical syndrome with multiple causes and contributions, characterized by decreased strength, resistance, and reduced physiological function that increases the vulnerability of individuals and leads to greater dependence and/or death”^(^ ^2^ ^)^.

The frailty syndrome in the elderly can be diagnosed according to the frailty phenotype, which consists of the measurement of five biological markers: gait velocity, hand grip strength, unintentional weight loss, level of physical activity, and self-report of fatigue/exhaustion. The elderly with three or more of these markers are considered frail; those with one or two criteria are in the pre-frail stage, and those who do not present any of the mentioned components are considered non-frail^(^ ^3^ ^)^.

Geriatric syndromes are multifactorial conditions that involve the interaction of stressing situations and risk factors that cause damage to the systems^(^ ^4^ ^)^. When associated with frailty, they represent a syndromic picture of a multisystemic nature^(^ ^3^ ^)^, which results in difficulty to restore functions and loss of independence^(^ ^5^ ^)^. This progression induces the decline of physiological functions until death^(^ ^3^ ^)^.

Recognized as one of the geriatric syndromes^(^ ^6^ ^)^, postural instability can be defined as the inability to integrate sensory information and determine body oscillations in the upright position while maintaining balance^(^ ^7^ ^)^. Although frequently used by researchers, there is no standardized use for the term postural balance, and for this reason, it is commonly used in association with other terms^(^ ^8^ ^)^.

“Balance involves the reception and integration of sensory stimuli, and the planning and execution of movements to control the center of gravity on the base support, by the postural control system that integrates information from the vestibular and somatosensory system and visual receptors”^(^ ^9^ ^–^ ^10^ ^)^.

During the aging process, these systems may become incapable of performing such functions. This leads to physical decline in the elderly and consequent impairment in the performance of daily tasks^(^ ^11^ ^)^. In a systematic review of the literature, researchers analyzed the predictive value of disabilities in activities of daily living (ADL) in the elderly and concluded that those with balance deficits were at greater risk of developing disabilities in ADL^(^ ^12^ ^)^. This functional limitation decreases postural control and predisposes the elderly to falls^(^ ^13^ ^)^.

According to the American Geriatrics Society (AGS) and the British Geriatrics Society^(^ ^14^ ^)^, falls are associated with restricted mobility, fractures, depression, functional disability, loss of independence and autonomy, institutionalization, decline in quality of life, socioeconomic implications, and over-burden to health systems.

Evidence shows that postural instability is related to frailty^(^ ^15^ ^–^ ^18^ ^)^ and pre-frailty^(^ ^19^ ^)^. In Brazil, a study conducted in Porto Alegre/Rio Grande do Sul with 521 elderly (≥ 60 years) received in the Primary Health Care (PHC) network associated the condition of frailty with geriatric syndromes. The frequency of postural instability was 36.5%. There was an association between frailty and postural instability (*p* < 0.001)^(^ ^20^ ^)^.

The literature review showed a small number of studies investigating the association between physical frailty and postural instability, and the studies mostly focused on community-dwelling elderly and assisted by PHC. The present study focuses on outpatient care. As this is an unexplored context, the study may provide new subsidies for the implementation of nursing care.

The scientific evidence on the relationship between the variables of interest of the study, as contributory elements to physical vulnerability, is essential to direct and consolidate nursing care actions for frail elderly people.

In view of the above, the objective of the present study was to analyze the relationship between postural instability and the condition and markers of physical frailty in elderly patients in geriatrics and gerontology outpatient care.

## Method

A cross-sectional study was carried out at a Geriatrics and Gerontology Outpatient Clinic (GGOC) in the city of São José dos Pinhais/PR (Brazil). The GGOC is a reference center and provides comprehensive care to all the elderly population (≥ 60 years old) residing in the municipality who uses the Unified Health System (SUS). The average monthly number of queries is approximately 300 queries/month.

The target population of the study corresponded to elderly people aged ≥ 60 years assisted in Primary Health Care, scheduled for consultation in the GGOC. In order to define a representative population sample, the total population of the elderly of São José dos Pinhais in the year 2015^(^ ^21^ ^)^ was taken as reference. A 95% confidence interval (CI) and a significance level of 5% (α = 0.05) were considered. The sample size included a margin of 8% for possible losses or refusals.

The selection of the participants was voluntary, and all the elderly were invited to participate in the research. Recruitment occurred randomly while the patients were waiting for consultation at the GGOC. The elderly were individually recruited after they first received information about the research and ethical aspects.

The criteria for inclusion of the elderly were: age ≥ 60 years; having attended the scheduled consultation in the GGOC; presence of cognitive ability, according to the results of the Mini Mental State Examination (MMSE)^(^ ^22^ ^)^. In turn, the exclusion criteria, according to observation of the medical records and/or notes from the medical consultation, were: presence of severe sequelae of stroke, with localized loss of muscle strength and aphasia; presence of neurological diseases that prevent the realization of the tests; presence of severe hearing or vision deficits that make communication difficult; physically inability to perform the proposed tests, and/or presence of upper or lower limb amputations; being in treatment for balance disorders with use of anti-dizziness medications.

A total of 411 elderly people were invited to participate in the study. Of these, one refused to participate and 29 were eliminated by the exclusion criteria. Therefore, the sample consisted of 381 elderly people.

Before starting data collection, the team of examiners provided a two-day training session of 8 hours for the support group. The group was made up of undergraduate nursing students (undergraduate scholarship holders) and students from the research group. The training aimed to standardize the collections, the applications of the tests, and the way of approaching the elderly in the outpatient clinic. During the data collection, the team and support group were coordinated by the researcher, that is, by the main author of the present manuscript.

A pilot study with ten elderly participants was carried out in order to check the adequacy of the instruments and make the necessary adaptations. There was no need for changes. Thus, the ten elderly participants were included in the sample. Data collection was carried out from September 2016 to March 2017.

The Mini Mental State Examination (MMSE)^(^ ^22^ ^)^ was employed for cognitive screening. The total score varies from zero to thirty, with the following cutoff points being adopted: “13 points for illiterate elderly; 18 points for those with low and medium schooling (one to eight incomplete years of study); and 26 points for high schooling (eight or more years of study)”^(^ ^23^ ^)^.

Data was collected using a sociodemographic questionnaire, an instrument for assessment of physical frailty, and for assessment of balance. The sociodemographic questionnaire covered the following variables: sex, age, marital status, schooling, race and monthly family income. The instrument was prepared and adapted according to the model of the Instituto Brasileiro de Geografia e Estatística (IBGE)^(^ ^24^ ^)^. Physical frailty was evaluated by means of the frailty phenotype^(^ ^3^ ^)^, as described below.

Hand grip strength (HGS) was measured in kilograms/force (Kgf) by means of a Jamar hydraulic dynamometer^®^, and followed the recommendation of the American Society of Hand Therapists (ASHT)^(^ ^25^ ^)^. The elderly performed three prehension movements in one-minute intervals to regain strength; the three values were recorded. The HGS values of each elderly participant were adjusted according to gender and Body Mass Index (BMI). Values that included the lowest quintile were considered markers of frailty^(^ ^3^ ^)^.

To evaluate gait speed (m/s), the elderly were instructed to walk a distance of 4.6 meters, usually on a flat surface. After adjusting for sex and height, the values in the lowest quintile were markers of frailty^(^ ^3^ ^)^.

Unintentional weight loss was checked through self-report of the participants in response to two questions: (1) “Have you lost weight in recent months?”; (2) “How many pounds?” The participants who reported loss of body weight greater than or equal to 4.5 kg in the last twelve months were considered frail for this marker, in an unintentionally manner (without diet or exercise)^(^ ^3^ ^)^.

Fatigue/exhaustion was assessed through the self-report of the participants, according to the participant's response to items 7 and 20 of the Center for Epidemiological Scale - Depression (CES-D)^(^ ^26^ ^–^ ^27^ ^)^, validated for community-dwelling Brazilian elderly^(^ ^28^ ^)^. Answers “2” or “3” for any of the questions indicated the elderly as frail for that marker^(^ ^3^ ^)^.

The Minnesota Leisure Activity Questionnaire^(^ ^29^ ^)^, validated for Brazilian elderly^(^ ^30^ ^)^, was used to assess level of physical activity. The questions relate to the frequency and time of activities carried out in the last year. The annual energy expenditure of each elderly person was calculated. After adjusting for sex, the values in the lowest quintile were considered indicators of frailty^(^ ^3^ ^)^.

Postural balance was evaluated through the Berg Balance Scale (BSE)^(^ ^31^ ^)^, translated and validated in Brazil^(^ ^32^ ^)^. This scale is aimed at frail elderly and evaluates functional balance with the objective of identifying the abilities and limitations to maintain balance during common activities of daily life. The cut-off point followed the recommendation, with scores < 45 indicating changes in balance and greater risk of falls^(^ ^31^ ^)^.

Data were organized and codified in the *Microsoft Excel* ^®^ 2007 software and analyzed in the statistical software R^®^ version 3.3.3. Univariate analyses were performed by the Chi-square test with a level of statistical significance of *p* ≤ 0.05, and multivariate analysis consisted of a Forward Stepwise logistic regression, which resulted in a model of physical frailty associated with postural instability.

The odds of each independent variable relating to postural instability were analyzed by Odds Ratio, with 95% CI. Each model was evaluated according to the Receiver Operating Characteristic Curve and predictive value, specificity and sensitivity; the model presenting the lowest value in the Akaike Information Criterion was considered eligible.

The research project was approved by the Ethics Committee of Research with Human Beings of the Health Sciences Sector of the institution, under Opinion CEP/SD 1.755.394 and CAAE: 58954016.1.0000.0102.

## Results

The results showed a homogeneous sample in relation to the variables gender and age group. The mean age of participants was 70.6 years (± 7.4), with a minimum of 60 years and a maximum of 100 years. The predominant characteristics of the participants were married marital situation (n = 251, 65.8%), one to four years of schooling (n = 206; 54%), white race (n = 310; 81.3%), and monthly family income of up to two minimum wages (n = 328, 86%).

As to the condition of physical frailty, 56 (14.7%) elderly were classified as frail, 217 (57%) as pre-frail, and 108 (28.3%) as non-frail. The most prevalent marker was decreased level of physical activity (n = 151, 39.6%), followed by self-report of fatigue and exhaustion (n = 98, 25.7%), slower gait speed (n = 77, 20.2%), reduced hand grip strength (n = 76, 19.4%), and unintentional weight loss (n = 62, 16.3%). It was found that 62 (16.3%) elderly had postural instability.

The condition of frailty was associated with postural instability (*p* = 0.000) and pre-frailty (*p* < 0.001), and with the following markers of physical frailty: reduced hand grip strength (*p* = 0.0008), unintentional weight loss (*p* = 0.0094), reduced level of physical activity (*p* = 0.0001), fatigue/exhaustion (*p* < 0.0001) and reduced gait speed (*p* < 0.0001) (Table 1).

**Table 1:** Association between postural instability and the condition and markers of physical frailty in the elderly. Curitiba - PR, Brazil

Condition of Physical frailty	Physical frailty	Postural instability		Total n (%)	p-value*
		Yes n (%)	No n (%)		
	Frail	33(58.9)	23(41.1)	56(100)	0.000
	Pre-frail	27(12.4)	190(87.6)	217(100)	<0.001
Markers of physical frailty	Reduced hand grip strength	22(8.9)	54(71.1)	76(100)	0.0008
	Unintentional weight loss	17(27.4)	45(72.6)	62(100)	0.0094
	Reduced level of physical activity	39(25.7)	113(74.3)	152(100)	0.0001
	Fatigue/exhaustion	37(37.8)	61(62.2)	98(100)	<0.0001
	Reduced gait speed	42(54.5)	35(45.5)	77(100)	<0.0001

* *p*-value < 0.05 obtained in the Chi-square test.

All markers of physical frailty were included in the construction of the predictive model of physical frailty associated to postural instability. The Forward Stepwise method allowed choosing a model that explained 87.9% of the variability of the data and which was satisfactory to predict physical frailty associated to postural instability. In order to choose the best model, the following variables were chosen: sensitivity (80.6%), specificity (80.2%) and accuracy (80.3%).

Table 2 shows the variables considered by the predictive model: reduced gait speed, self-report of fatigue/exhaustion, reduced level of physical activity, and unintentional weight loss. The model indicates that these markers, when present, significantly increase postural instability. The odds of postural instability increased in the elderly with reduced gait speed (OR = 14.58; 95% CI: 7.34-30.18), fatigue/exhaustion (OR = 5.45; 95% CI: 2.72-11.27); reduced level of physical activity (OR = 2.47, 95% CI: 1.24-5.02), and unintentional weight loss (OR = 2.00, 95% CI: 0.87-4.51).

**Table 2:** Final logistic regression model associated with the postural instability of elderly people. Curitiba - PR, Brazil

Markers of physical frailty	Estimate	Standard error	Z Test Estimate	p-value*	OR†	95% CI†
Reduced gait speed	22(8.9)	54(71.1)	76(100)	0.0008	14.58	7.34 – 30.18
Fatigue/exhaustion	17(27.4)	45(72.6)	62(100)	0.0094	5.45	2.72 – 11.27
Reduced level of physical activity	39(25.7)	113(74.3)	152(100)	0.0001	2.47	1.24 – 5.02
Unintentional weight loss	37(37.8)	61(62.2)	98(100)	<0.0001	2.00	0.87 – 4.51

* *p*-value < 0.05 obtained in the Wald test;

† OR - *Odds Ratio*;

‡ CI - 95% confidence interval. All information was obtained after adjustment of the logistic regression evaluated by the Akaike Information Criterion via Stepwise Forward method.

## Discussion

The condition of physical pre-frailty was observed in more than half of the sample. In contrast with international^(^ ^3^ ^,^ ^33^ ^)^ and national^(^ ^34^ ^)^ studies, the values of frailty and pre-frailty found here were significantly higher. These investigations present different results from the present study, as they were conducted with community-dwelling elderly whose characteristics differ from those of elderly in the outpatient context.

International studies show great variability in the frequency of physical frailty, either in homogeneous or different populations of elderly people^(^ ^35^ ^)^. Authors of the prospective observational Cardiovascular Health Study (CHS) conducted in the United States of America (USA), worked with an initial cohort of 5,201 elderly people aged 65 to 101 years and then received another cohort of 687 elderly. The percentage of frailty ranged from 7 to 12%^(^ ^3^ ^)^.

Researchers point to socio-demographic and economic differences of developed countries to explain the variations in prevalence values of the syndrome ^(^ ^35^ ^)^. Such variability is noticed boths in the conditions of physical frailty and also in its markers.

In eleven European countries (Sweden, Denmark, Germany, Netherlands, Belgium, Switzerland, Austria, France, Italy, Spain and Greece), frailty was present in 8.8% of the people aged over 50 years, and pre-frailty in 39.1%^(^ ^33^ ^)^. In turn, in South America (developing countries), the prevalence of frailty was reported to be 19.6%^(^ ^36^ ^)^ and in Brazil 9.0%, according to data from the Frailty in Brazilian Elderly (FIBRA) study^(^ ^34^ ^)^.

In the present investigation, decreased level of physical activity, fatigue/exhaustion, and reduced gait speed were markers related to muscle strength and poor physical activity. The frequency distribution of markers of physical frailty varies between studies according to characteristics of the samples; however, the predominant markers are always related to aspects of strength and physical activity^(^ ^37^ ^–^ ^38^ ^)^. It was found that, a considerable part of the participants of the present study did not practice any physical activity.

Researchers from a cross-sectional study developed in the city of Limburg/Maastricht (Netherlands) with 8,864 elderly (≥ 65 years) compared the condition and markers of physical frailty to various health domains (social, psychological and physical). The more significant markers identified in this study differ from those found here: reduced gait speed (16%) and decreased level of physical activity (13%)^(^ ^37^ ^)^. The percentage of decreased hand grip strength (20%) is in line with the results of the present investigation.

Another cross-sectional study developed in Quebec (Canada) associated the markers of physical frailty to the basic and instrumental activities of the daily living of 1,643 community-dwelling elderly (≥ 65 years). The predominant marker was reduced gait speed (20.1%) followed by self-report of fatigue/exhaustion (19.2%) and decreased level of physical activity (14.2%)^(^ ^11^ ^)^. The domain of the markers was similar to that obtained in this study.

In Brazil, researchers from the FIBRA study in fourteen cities evaluated the contribution of each marker in the determination of frailty. In the sample of 5,532 community-dwelling elderly (≥ 65 years), the prevalent markers were decreased level of physical activity (27.5%), reduced gait speed (20.9%), and hand grip strength (20.6% %)^(^ ^38^ ^)^.

The frailty markers identified as most significant in the sample^(^ ^38^ ^)^ corroborate the results of the present study. There was a significant difference in the percentage of reduced level of physical activity.

Regarding postural instability, the value found in the present study was lower when compared to the percentages of international^(^ ^39^ ^)^ and national^(^ ^40^ ^–^ ^41^ ^)^ studies. In these studies we found a diversity of terms and concepts for the syndrome of postural instability, as well as disagreement between their results.

A cross-sectional research using the database of the Health Interview Survey in Ann Arbor/Michigan, USA, analyzed the prevalence and types of dizziness among the American population. Of 33.4 million respondents, 14.8% reported problems with dizziness or imbalance in the last 12 months. The balance problems were: postural instability (61.3%), dizziness (49%), fainting (40.8%), vertigo (36.8%), fluctuation (25%) and changes in the vision during head rotation (24%). The authors concluded that the population reported several types of dizziness^(^ ^39^ ^)^. The findings showed this diversity of terms and concepts used.

The term dizziness is widely used in developing countries. Researchers of the cross-sectional study developed by the FIBRA network with a sample of 391 elderly individuals (≥ 65 years) analyzed the relationship between dizziness, sociodemographic factors, diseases, and geriatric syndromes. Dizziness during the last year was reported by 176 (45%) elderly. For the authors, dizziness is considered a geriatric syndrome due to its substantial prevalence, with a nonspecific and complex manifestation in the elderly population^(^ ^40^ ^)^.

Higher values were found in a population-based research developed in Belo Horizonte/Minas Gerais (Brazil) which investigated the prevalence of dizziness according to the Household Sample Survey (PAD-MG). Of the 19,442,971 million people investigated, 18.4% presented some health problem. Dizziness was the third major complaint; 48.3% of the participants reported feeling dizziness in the last month. The distribution among the elderly (≥ 60 years) was 34.8%^(^ ^41^ ^)^.

In the city of São Paulo (Brazil), the prevalence of dizziness was 42%, with a 44% increase in the elderly population (≥ 65 years)^(^ ^42^ ^)^. In Cuiabá/Mato Grosso (Brazil), 45% of the elderly reported dizziness, and approximately 70% imbalance or instability^(^ ^40^ ^)^. This variation of prevalence found between cities can be influenced by methodological biases such as the configuration of data collection, the description of the symptom, and the measure of prevalence used, with consequent increase of the values found.

In the present study, all markers of physical frailty were associated with postural instability: hand grip strength, unintentional weight loss, physical activity, fatigue/exhaustion, and gait speed. Furthermore, there was an association between postural instability and the conditions of pre-frailty and frailty.

Regarding the statistical association between postural instability and frailty, studies conducted in the USA^(^ ^19^ ^)^, France^(^ ^18^ ^)^ and even in Brazil^(^ ^20^ ^,^ ^43^ ^)^ reinforce the results of the present study. In the community of Arizona/Tucson (USA), with 125 elderly (≥ 65 years), researchers evaluated gait, balance and physical activity as possible markers of physical frailty. Inertial sensors in the body were used to evaluate the balance, and the frailty phenotype to classify the condition of physical frailty. The elderly were considered frail, 16.8% (n = 21), pre-frail, 48% (n = 60), and non-frail, 35% (n = 44). The findings showed that balance is a specific marker of pre-frailty (OR = 1.12; 95% CI: 1.05 to 1.20)^(^ ^19^ ^)^.

In this sense, researchers in Troyes/Champagne (France) investigated the relationship between balance and physical frailty of 186 elderly people in the community (≥ 65 years). Balance was evaluated by the *Balance Quality Tester* and frailty by the frailty phenotype. There were 12.9% (n=24) frail, 52.7% (n = 98) pre-frail, and 34.4% (n = 64) non-frail elderly individuals. There was an association between balance and frailty (*p* < 0.05)^(^ ^18^ ^)^.

In the Brazilian context, a cross-sectional study conducted in Porto Alegre/Rio Grande do Sul investigated 521 elderly (≥ 60 years) assisted in the Primary Health Care. The objective was to associate the frailty condition with geriatric syndromes. There were 21.5% frail, 51.1% pre-frail, and 27.4% robust elderly. They only preceded the frequency of postural instability (36.5%), cognitive decline (54.7%), and polypharmacy (41.2%). There was association between postural instability and frailty (*p* < 0.001)^(^ ^20^ ^)^.

In order to evaluate the balance and classify the elderly as frail, non-frail and pre-frail, an intervention study developed in the city of Ribeirão Preto/São Paulo (Brazil) investigated 60 elderly individuals (≥ 65 years). Balance was evaluated by the BESTest and with a force platform. The result of such study were in line with those of the present study, pointing out that balance is lower in frail elderly 14.2% (n = 60) when compared to the non-frail ones (*p* = 0.0001)^(^ ^44^ ^)^.

It is emphasized that postural instability in elderly patients is worrisome due to the consequences of this clinical characteristic, which are falls. In Goiânia/Goiás (Brazil), researchers evaluated an intervention program for the prevention of falls in a randomized controlled clinical trial with 20 institutionalized elderly (≥ 60 years). After 12 months of physical exercise intervention, the results showed a reduction in the number of falls (*p* = 0.046), improvement in balance (*p* = 0.001), balance and gait (*p* = 0.007), hand grip strength (*p* = 0.001), lower limbs (*p* < 0.001), and shoulder flexion (*p* = 0.001)^(^ ^45^ ^)^.

The predictive model of physical frailty for the elderly associated to postural instability was considered satisfactory. The chances of postural instability increased in elderly individuals with reduced gait speed, self-report of fatigue/exhaustion, lower level of physical activity, and unintentional weight loss. The use of the predictive model favors the clinical reasoning and provides subsidies for the objective and specific clinical practice.

The association between postural instability and gait speed reduction and the high chance of elderly individuals with the markers reduced gait speed to present postural instability is noteworthy. The management of care in elderly patients is based on the evaluation of gait speed. Such trait can be easily measured in the elderly and does not imply costs^(^ ^46^ ^)^. Continuous evaluation provides support for the prevention of falls and improves gerontological professional practice. Several studies define this component as the main marker of the condition of physical frailty^(^ ^46^ ^–^ ^48^ ^)^.

In Curitiba/Paraná (Brazil), an intervention study with 62 elderly subjects (≥ 60 years) evaluated and compared the muscular strength, kinematic gait parameters, and performance in functional tests of elderly individuals with or without a history of falls. The participants were divided into two groups, and speed was one of the gait parameters evaluated. There was no association between variables and falls (*p* = 0.06; OR = 2.30; 95% CI: 0.95-5.59), and gait speed was lower among elderly who had falls compared to those who did not have a history of falls. The researchers concluded that balance is a protective factor against falls^(^ ^49^ ^)^.

An important marker for maintaining balance is the practice of physical activity according to the results of a cohort study developed in Ulm/Baden-Württemberg (Germany) with 1,271 community-dwelling elderly people (≥ 65 years). The average duration of daily walking was 104.8 minutes for men, and 103.0 minutes for women. Balance was related to the average duration of daily walking in men (OR = 24.3; 95% CI: 17.8-30.9) and women (OR = 17.4; 95% CI: 11.8-23.0)^(^ ^50^ ^)^.

The predominance of reduced level of physical activity and the association with the physical frailty of the elderly reinforces the recognition that this parameter is an important marker of frailty. It is important to emphasize that the nursing professionals should encourage the elderly to daily physical activity, because its benefits in the aging process are a consensus in the current literature^(^ ^51^ ^)^.

A longitudinal study was conducted for three years in Suwon/South Korea. The researchers investigated the influence of frailty and BMI category on mortality in elderly people in 11,844 community-dwelling Koreans (≥ 65 years). They were classified as frail 7.8%, pre-frail 50.4%, and non-frail 41.8%. Frailty was associated with low weight and risk of mortality (OR = 8.81; 95% CI: 5.00-15.5). According to the authors, BMI may represent reduced reserve capacity, weight loss and consequently, more adverse outcomes^(^ ^52^ ^)^. It is worth noting that in the present study, the values found were lower (OR = 2.00; 95% CI: 0.87-4.51).

In the city of Abu/Nagoya (Japan), a prospective cohort study followed-up 4,341 elderly (≥ 65 years) for two years and identified the frailty components with the greatest impact on the elderly's disability. The results showed that slowness (OR = 2.32; 95% CI: 1.62-3.33), weakness (OR = 1.90; 95% CI: 1.35-2.68), and weight loss (OR = 1.61; 95% CI: 1.13-2.31) were strongly associated with disability^(^ ^53^ ^)^. These results are similar to the present study, although divergent in the marker reduced level of physical activity.

Managing physical frailty^(^ ^2^ ^)^ in frail elderly, especially in the outpatient setting, recommends that health professionals adopt measures including the dissemination of resolutive interventions, which are effective when developed at gerontological and geriatric levels, that is, interdisciplinarily. The attentive look of nurses at the elderly's physical activity, reduction of polypharmacy, adequate intake of vitamin D, and adequate caloric-protein support should boost the management of frailty and, consequently, minimize the effects of postural instability.

The study had some limitations regarding the methodological design of the cross-sectional type, which makes it impossible to evaluate causes and effects. Another important limitation was the diversity in the definition of the term postural instability seen in several studies, scales and descriptors in health sciences. This dissimilarity was an inconvenience throughout the research process, hindering the location of studies in the current literature and the discussion of the data.

It is considered that the use of some data collection instruments consisting of self-report questions for evaluation of physical frailty markers is likely to generate biases in the results. Moreover, the instrument used to measure physical activity (the Minessota Leisure Time Activities Questionnarie) includes unusual types of physical activity in the Brazilian context.

## Conclusion

There was a significant association between postural instability and the condition and markers of physical frailty. The presence of postural instability determines a greater chance of the elderly being categorized as frail or pre-frail. The predominance of reduced level of physical activity reinforces the recognition that physical activity is an important marker of frailty.

The predictive model of physical frailty associated with postural instability was considered satisfactory and indicated that the following markers, when present, significantly increased postural instability: reduced gait velocity, fatigue/exhaustion, decreased level of physical activity, and unintentional weight loss.

In clinical practice, gerontological nursing should ensure continuous standards of evaluation of these markers and recognize relative risks to favor the management of physical frailty among health professionals and in the elderly.

The results of the present study bring significant contributions to gerontological practice and reinforce the need for nursing to create effective opportunities for older people to engage with physical activity practices.
